# Prognostic Factors of Physical Function Decline Among Middle-Aged Adults With HIV

**DOI:** 10.1093/ofid/ofaf311

**Published:** 2025-05-27

**Authors:** Grace L Kulik, Triin Umbleja, Todd T Brown, Heather J Ribaudo, Steven K Grinspoon, Jennifer A Schrack, Markella V Zanni, Marissa R Diggs, Judith A Aberg, Carl J Fichtenbaum, Carlos D Malvestutto, Sarah M Chu, Judith S Currier, Pamela S Douglas, Gerald S Bloomfield, Alice C Thornton, Michelle A Floris-Moore, Elliot Goodenough, Grant B Ellsworth, Tricia Burdo, Kristine M Erlandson

**Affiliations:** University of Colorado Anschutz Medical Campus, Aurora, Colorado, USA; Harvard TH Chan School of Public Health, Boston, Massachusetts, USA; Johns Hopkins University School of Medicine, Baltimore, Maryland, USA; Harvard TH Chan School of Public Health, Boston, Massachusetts, USA; Massachusetts General Hospital and Harvard Medical School, Boston, Massachusetts, USA; Johns Hopkins Bloomberg School of Public Health, Baltimore, Maryland, USA; Massachusetts General Hospital and Harvard Medical School, Boston, Massachusetts, USA; Massachusetts General Hospital and Harvard Medical School, Boston, Massachusetts, USA; Icahn School of Medicine at Mount Sinai, New York, New York, USA; University of Cincinnati College of Medicine, Cincinnati, Ohio, USA; Ohio State University Medical Center, Columbus, Ohio, USA; Massachusetts General Hospital and Harvard Medical School, Boston, Massachusetts, USA; David Geffen School of Medicine, University of California, Los Angeles, California, USA; Duke University Research Institute, Duke University School of Medicine, Durham, North Carolina, USA; Duke University Research Institute, Duke University School of Medicine, Durham, North Carolina, USA; Duke Global Health Institute and Duke Clinical Research Institute, Duke University, Durham, North Carolina, USA; University of Kentucky College of Medicine, Lexington, Kentucky, USA; University of North Carolina School of Medicine, Chapel Hill, North Carolina, USA; Drexel University College of Medicine, Philadelphia, Pennsylvania, USA; Weill Cornell Medicine, New York, New York, USA; Temple University Lewis Katz School of Medicine, Philadelphia, Pennsylvania, USA; Rutgers Biomedical Health and Science, University Rutgers University, New Brunswick, New Jersey, USA; University of Colorado Anschutz Medical Campus, Aurora, Colorado, USA

**Keywords:** aging, functional impairment, HIV, physical function, screening

## Abstract

**Background:**

Pitavastatin to REduce Physical Function Impairment and FRailty in HIV (PREPARE) found small declines in physical function overall among people with HIV (PWH). However, there was substantial individual variability. The purpose of this prespecified exploratory analysis was to identify the PWH at greatest risk for physical function decline.

**Methods:**

Participant-specific annualized rates of change on annually measured chair rise rate, gait speed, the modified Short Performance Physical Battery (composite of the latter 2 plus balance time), and grip strength were estimated from linear mixed-effect models. Change in performance that was below the 20th percentile of the study population in ≥1 measure was classified as physical function decline. Associations between baseline factors and physical function decline were evaluated with log-binomial regression models.

**Results:**

Of 569 participants (81% male, 52% White), the median age (Q1–Q3) was 51 (47–55) years. Half (52%) of the participants had decline in physical function. The risk of decline was higher among females (relative risk [RR], 1.32; 95% CI, 1.12–1.55) and non-Whites (RR, 1.23; 95% CI, 1.05–1.45) and tended to increase with age (50–55 years: RR, 1.04; 95% CI, 0.86–1.26; 55+ vs 40–<50 years: RR, 1.17; 95% CI, 0.98–1.39). In models adjusted for age, sex, and race, we found greater risk of decline among those with history of depression treatment, higher body mass index (BMI), preexisting functional impairment, frailty (by index), and higher baseline high-sensitivity C-reactive protein and interleukin-6 levels.

**Conclusions:**

PWH with history of depression treatment, high BMI, or levels of inflammation and those showing early signs of functional impairment may be at higher risk of physical function decline and should be targeted for early interventions to preserve physical function with aging.

## Teaser

We examined factors associated with physical function decline among a middle-aged adults with HIV in a substudy of REPRIEVE. Those with a history of depression treatment, high body mass index or high levels of inflammation, and those with pre-existing functional impairment were at greater risk for physical function decline with aging.

Preservation of physical function is an essential component of healthy aging [1]. Persons with HIV (PWH) appear to have an accelerated decline of physical function compared with populations without HIV, which may contribute to increased morbidity and all-cause mortality [2]. Many factors may contribute to this faster physical function decline, including chronic inflammation, adverse effects from antiretroviral use, social determinants of health, and greater comorbidity burden [3, 4].

The rate of physical function decline among PWH is heterogenous: Many PWH maintain a high level of function with increasing age, while others have a particularly rapid decline. Identifying factors underlying this heterogeneity is essential in identifying targets for interventions, as well as identifying populations who may benefit most from interventions to slow decline or preserve physical function later into life [5]. Similarly, earlier detection can guide preventative strategies to maximize function [5]. Prior studies examining physical function decline among PWH were conducted in cohorts that were older, had more chronic conditions, and/or did not have viral suppression [6, 7]. However, identifying factors associated with physical function decline occurring earlier in life is particularly important, as midlife may provide a more effective window for intervention.

The Pitavastatin to REduce Physical Function Impairment and FRailty in HIV (PREPARE), ancillary study of Randomized Trial to Prevent Vascular Events in HIV (REPRIEVE), recently examined the impact of statins on longitudinal change in physical function among PWH [8–10]. Primary findings from PREPARE showed minimal declines in physical function overall, and no impact of pitavastatin on the rate of change in physical function. Despite minimal overall decline in physical function, there was substantial individual heterogeneity in physical function decline [10]. The purpose of this prespecified exploratory analysis was to identify participants who experienced declines in physical function and evaluate baseline factors (including demographics, lifestyle and clinical characteristics, markers of inflammation and immune activation) associated with this decline.

## METHODS

### Study Design

This exploratory analysis utilized data from REPRIEVE (NCT02344290) participants who enrolled in the physical function ancillary study PREPARE (NCT03070223), a prospective observational longitudinal cohort study. REPRIEVE participants were randomized to receive pitavastatin calcium or placebo. Those enrolled in the United States were eligible to enter PREPARE within 24 months of REPRIEVE entry, with follow-up for 5 years from REPRIEVE entry [11, 12]. The present analysis was restricted to participants with physical function assessments available at 2 or more time points for all physical function measures to facilitate evaluation of participant-specific declines over time. Physical function assessments were performed annually.

### Study Participants

REPRIEVE recruited men and women with HIV, between 40 and 75 years old, receiving stable ART, with a CD4+ T-cell count >100 cells/mm^3^, and with low to moderate traditional cardiovascular disease (CVD) risk, as represented by the pooled cohort Atherosclerotic Cardiac Risk Score (ASCVD) [11]. Key exclusion criteria included known CVD, current diabetes mellitus if LDL cholesterol ≥70 mg/dL, active cancer, impaired kidney function, chronic hepatitis with significant liver fibrosis, and myositis or myopathy. Additional inclusion and exclusion criteria have been previously described [11]. PREPARE recruited at the ACTG US sites participating in REPRIEVE from March 2017 to February 2018.

### Physical Function Measures

Physical function assessments were conducted at each participating site by centrally trained personnel. Physical function was assessed by the composite score of the modified SPPB (mSPPB) [13] and grip strength. Grip strength was calculated as the average of 3 measurements. The mSPPB tests included a 10× chair rise, 4-meter walk at usual pace measured twice, and balance (semitandem, tandem pose, and single-leg balance). Composite mSPPB score (ranging from 0 to 3 = maximal performance) was calculated as a sum of the following 3 components each divided by the maximal possible performance to derive a ratio between 0 and 1: (1) chair rise rate divided by maximal performance of 1 stand/second; (2) proportion of total standing balance time calculated as the total time each of the semitandem, tandem, and 1-leg stand positions was held (maximum 30 seconds each) divided by 90 seconds; and (3) gait speed based on the average of 2 walk times divided by maximal performance of 2 m/s [13]. For all outcome measures, participants unable to attempt the test and tests not attempted for nonadministrative reasons were assigned the worst score. Participants were considered to have physical function decline if their individual annualized rate of change fell below the 20th percentile of the study population in any of the physical function measures. This threshold was selected in accordance with how the criteria for frailty are operationalized in large population studies [14].

### Covariates

Demographic and clinical data were obtained at enrollment as previously described [15]. Factors to evaluate in relation with physical function decline were chosen a priori and included baseline demographics of age, natal sex, and race. For females, postmenopausal status was defined as no menses within 12 months or double oophorectomy. Behavioral characteristics included self-reported cigarette smoking status, substance use (cocaine, methamphetamine, injection drugs), alcohol use, physical activity (based on frequency of doing <30 minutes of physical activity 3 or more days a week), and having >2 hours of screen time per day (Rapid Eating Assessment for Patients [REAP] questions 26 and 27). Clinical and cardiometabolic factors included body mass index (BMI; calculated as body weight in kilograms/height in meters squared), metabolic syndrome, hypertension, ASCVD risk score, having ever been treated for depression with medications per self-report, and self-reported presence of muscle aches or weakness. HIV characteristics included history of AIDS-defining event, cumulative years of ART use, history of thymidine analog exposure, current CD4+ cell count, and HIV-1 RNA suppression. Preexisting functional impairment was defined as scoring 34.7–58.2 (some impairment) or <34.7 (moderate to severe impairment) on the Duke Activity Status Index (DASI) [16]. Frailty was defined using the Frailty Index with a score of >0.2 [17]. Blood was obtained after fasting at the enrollment visit for biomarkers, including high-sensitivity C-reactive protein (hsCRP), monocyte chemoattractant protein–1 (MCP-1), high-sensitivity interleukin-6 (IL-6), interleukin-10 (IL-10), soluble tumor necrosis factor receptor–1 (sTNFR-1), soluble CD14 (sCD14), soluble CD163 (sCD163), growth differentiation factor 15 (GDF-15), fibroblast growth factors 21 and 23 (FGF-21, FGF-23), and cellular biomarkers %CD38+;HLADR+ on CD4+ and %CD38+;HLADR+ on CD8+ and %CD14+;CD16+ (inflammatory) on monocytes. Biomarkers were measured from plasma using enzyme-linked immunosorbent assay kits (R&D Systems) except for IL-10 (Meso Scale Discovery, MSD V-PLEX platform) in the laboratory of Dr. Tricia Burdo at Temple University, Philadelphia, Pennsylvania, USA [18].

### Statistical Analysis

Participant-specific annualized rates of change in each of the 4 physical function outcome measures were estimated using linear mixed-effect models including random intercept and slope, following the PREPARE primary analytic approach and assumptions as previously described [10]. Time was modeled as continuous (with REPRIEVE entry as 0). Change in physical function was modeled as linear in time, and, given no treatment effect observed in the PREPARE primary analysis [10], treatment group was not included in the models.

Risk factors to evaluate in relation to physical function were screened based on the Spearman correlation coefficients for associations with the participant-specific slopes in each of the 4 physical function outcome measures (continuous), as well as with the composite physical function decline (binary). Factors with *P* values <.1 and a consistent pattern across outcome measures were evaluated further in log-binomial regression models with the composite physical function decline as the outcome. Each factor was evaluated individually (unadjusted), and adjusting for age, natal sex, and race (known to be associated with physical function and many of the factors examined). In an ad hoc analysis, the models for sex effect were further expanded to examine whether the effect may be explained by differences between males and females. Results are presented as forest plots of estimated relative risks (RRs) of physical function decline with 2-sided 95% CIs, and type 3 *P* values are shown. Inference is generally based on CIs and 2-sided *P* values <.05, with no adjustment for multiplicity in this exploratory analysis. The analyses were conducted using SAS software (version 9.4 for Linux; SAS Institute Inc., Cary, NC, USA).

## RESULTS

### Participant Characteristics and Follow-up

Of the 602 participants enrolled in PREPARE, 569 had physical function assessments available at 2 or more time points for all physical function measures and were included in the present analysis. The cohort was primarily male, 52% White, 39% Black, and 18% Hispanic or Latino ethnicity, with a median (Q1, Q3) age of 51 (47, 55) years (Table 1). Current smoking was reported by 27%, and current substance use by 2%. Participants were relatively healthy, generally without major comorbidities, with relatively high physical function as previously reported [10, 12], and 94% had HIV viral suppression. The study follow-up and CONSORT were previously published [10]; an additional 32 participants (n = 17 in the pitavastatin group and 15 in the placebo group) who had a single evaluation of physical function were excluded from this analysis because their individual rate of change in physical function could not be estimated.

**Table 1.** Baseline Demographics by Physical Function Decline and by Natal Sex

Characteristic		Physical Function		Total (n = 569), No. (%)	Natal Sex	
		Decline (n = 297), No. (%)	No Decline (n = 272), No. (%)		Male (n = 462), No. (%)	Female (n = 107), No. (%)
Treatment group	Pitavastatin	151 (51)	148 (54)	299 (53)	237 (51)	62 (58)
	Placebo	146 (49)	124 (46)	270 (47)	225 (49)	45 (42)
Physical function decline		297 (100)	0 (0)	297 (52)	224 (48)	73 (68)
Age, y	40–44	39 (13)	41 (15)	80 (14)	66 (14)	14 (13)
	45–49	69 (23)	71 (26)	140 (25)	113 (24)	27 (25)
	50–54	93 (31)	91 (33)	184 (32)	149 (32)	35 (33)
	55–59	71 (24)	48 (18)	119 (21)	97 (21)	22 (21)
	60+	25 (8)	21 (8)	46 (8)	37 (8)	9 (8)
Natal sex	Male	224 (75)	238 (88)	462 (81)	462 (100)	NA
	Female	73 (25)	34 (13)	107 (19)	NA	107 (100)
Gender identity	Cisgender	292 (98)	262 (96)	554 (97)	448 (97)	106 (99)
	Transgender Spectrum	3 (1)	8 (3)	11 (2)	11 (2)	0 (0)
	Not reported	2 (1)	2 (1)	4 (1)	3 (1)	1 (1)
Menopausal status^a^	Postmenopausal	41 (57)	19 (56)	60 (57)	-	60 (57)
	Not postmenopausal	27 (37)	13 (38)	40 (38)	-	40 (38)
	Indeterminate	4 (6)	2 (6)	6 (6)	-	6 (6)
Race^b^	White	135 (45)	161 (59)	296 (52)	269 (58)	27 (25)
	Black or African American	137 (46)	87 (32)	224 (39)	153 (33)	71 (66)
	Asian	1 (0)	4 (1)	5 (1)	4 (1)	1 (1)
	Other	24 (8)	20 (7)	44 (8)	36 (8)	8 (7)
Ethnicity	Hispanic or Latino	51 (17)	52 (19)	103 (18)	84 (18)	19 (18)
	Not Hispanic or Latino	244 (82)	217 (80)	461 (81)	373 (81)	88 (82)
	Unknown	2 (1)	3 (1)	5 (1)	5 (1)	0 (0)
Smoking status	Current	94 (32)	60 (22)	154 (27)	125 (27)	29 (27)
	Former	89 (30)	87 (32)	176 (31)	145 (31)	31 (29)
	Never	114 (38)	125 (46)	239 (42)	192 (42)	47 (44)
Physical activity	Ideal	27 (9)	35 (13)	62 (11)	54 (12)	8 (8)
	Intermediate	159 (54)	149 (55)	308 (54)	258 (56)	50 (47)
	Poor	110 (37)	86 (32)	196 (35)	148 (32)	48 (45)
CD4 count, cells/mm³	Median (Q1, Q3)	610 (436, 785)	626 (487, 841)	615 (458, 800)	609 (456, 789)	646 (479, 1010)
HIV-1 RNA, copies/mL	<50	268 (93)	253 (94)	521 (94)	426 (93)	95 (96)
	≥50	19 (7)	15 (6)	34 (6)	30 (7)	4 (4)
ASCVD risk score, %	Median (Q1, Q3)	5.1 (2.8, 8.0)	4.4 (2.5, 6.6)	4.7 (2.7, 7.3)	5.2 (3.3, 7.9)	2.4 (1.2, 4.2)
BMI, kg/m²	Median (Q1, Q3)	28.1 (24.8, 31.0)	26.5 (23.8, 29.1)	27.2 (24.3, 30.2)	26.7 (24.0, 29.3)	30.4 (25.9, 35.5)
Hypertension		116 (39)	94 (35)	210 (37)	162 (35)	48 (45)
Depression treatment history		154 (52)	116 (43)	270 (47)	210 (45)	60 (56)
Muscle aches/weakness		65 (22)	45 (17)	110 (19)	81 (18)	29 (27)
hs-CRP, mg/L^c^	Median (Q1, Q3)	2.1 (1.0, 4.3)	1.3 (0.7, 2.7)	1.7 (0.8, 3.5)	1.6 (0.8, 3.0)	2.8 (1.3, 5.3)
sTNFR1, pg/mL^c^	Median (Q1, Q3)	1042 (869, 1238)	1025 (853, 1195)	1029 (865, 1205)	1016 (850, 1201)	1065 (951, 1281)
IL-6, pg/mL^c^	Median (Q1, Q3)	1.9 (1.1, 3.0)	1.4 (0.9, 2.4)	1.6 (1.0, 2.7)	1.5 (1.0, 2.6)	2.0 (1.2, 3.2)
sCD14, ng/mL^c^	Median (Q1, Q3)	1800 (1533, 2123)	1831 (1538, 2136)	1822 (1533, 2134)	1756 (1502, 2120)	1937 (1593, 2187)
sCD163, ng/mL^c^	Median (Q1, Q3)	883 (653, 1117)	833 (630, 1048)	854 (646, 1080)	846 (633, 1074)	870 (681, 1121)
%CD14+;CD16+ (inflammatory)^c^	Median (Q1, Q3)	7.3 (5.4, 9.3)	6.3 (4.6, 8.7)	6.9 (5.0, 8.9)	6.7 (4.8, 8.6)	7.8 (6.1, 10.6)
GDF-15, pg/mL^c^	Median (Q1, Q3)	827 (549, 1095)	773 (575, 1006)	810 (562, 1052)	811 (562, 1052)	807 (562, 1040)
Impairment by DASI	None	183 (62)	201 (74)	384 (67)	333 (72)	51 (48)
	Some	81 (27)	56 (21)	137 (24)	99 (21)	38 (36)
	Moderate–severe	33 (11)	15 (6)	48 (8)	30 (6)	18 (17)
Frailty status by index	Nonfrail	163 (55)	187 (69)	350 (62)	297 (64)	53 (50)
	Prefrail	111 (37)	79 (29)	190 (33)	146 (32)	44 (41)
	Frail	23 (8)	6 (2)	29 (5)	19 (4)	10 (9)

Missing data include menopausal status (n = 1), physical activity (n = 3), HIV-1 RNA (n = 14), hs-CRP (n = 96), sTNFR1 and GDF-15 (n = 137), IL-6, sCD14, and sCD163 (n = 95), %CD14+;CD16+ (inflammatory; n = 105).

Abbreviations: BMI, body mass index; DASI, Duke Activity Status Index; GDF-15, growth differentiation factor 15; hs-CRP, high-sensitivity C-reactive protein; IL-6, interleukin 6; RR, relative risk; sCD14, soluble CD14; sCD163, soluble CD163; sTNFR1, soluble tumor necrosis factor receptor–1.

^a^Menopausal status is summarized among females (n = 107).

^b^‘Other” race includes participants self-identifying as: native or indigenous to the enrollment region, more than 1 race (with no single race noted as predominant), or of unknown race.

^c^Biomarkers were tested as part of the REPRIEVE Mechanistic Substudy; they are not available for PREPARE participants not co-enrolled in the REPRIEVE Mechanistic Substudy.

### Participant-Specific Changes in Physical Function

**Figure 1.**
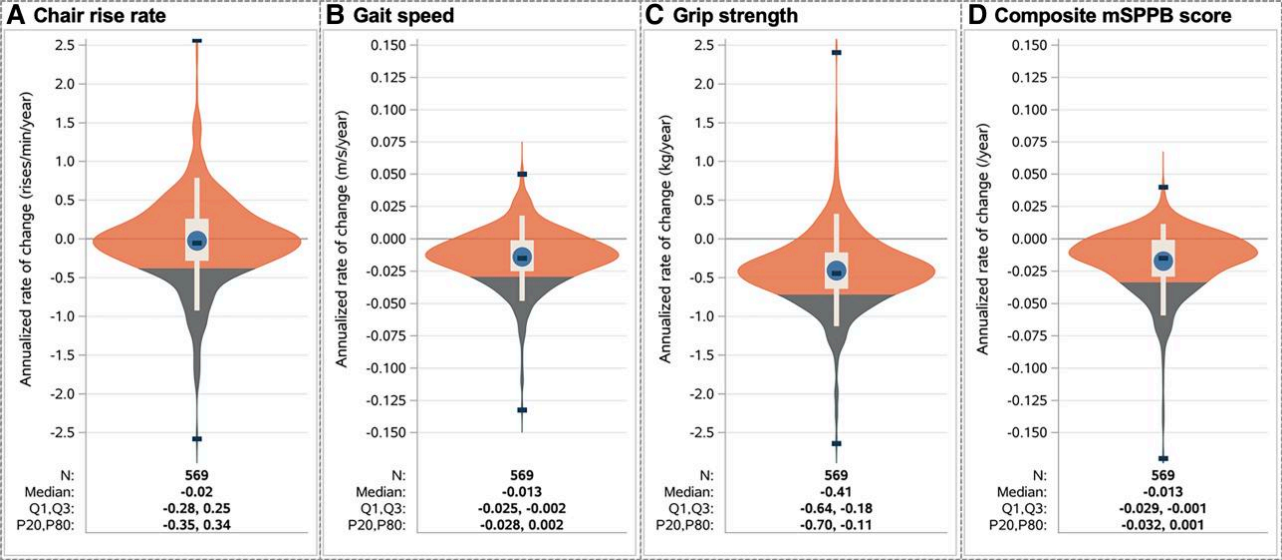
Participant-specific annualized rates of change in physical function. Violin plots showing Kernel estimate for probability density function and mean (circle), median, minimum and maximum (dashes), Q1–Q3 (box), and P5–P95 (whiskers); density below the 20th percentile is shown in the gray shading—these are the participants classified as having had a physical function decline. On the y-axis, 0 represents no change, values >0 improving, and values <0 declining physical function over follow-up. **A)** represents chair rise rate, **B)** represents gait speed, **C)** represents grip strength, and **D)** represents compositive SPPB score. Abbreviation: mSPPB, modified Short Physical Performance Battery.

**Figure 2.**
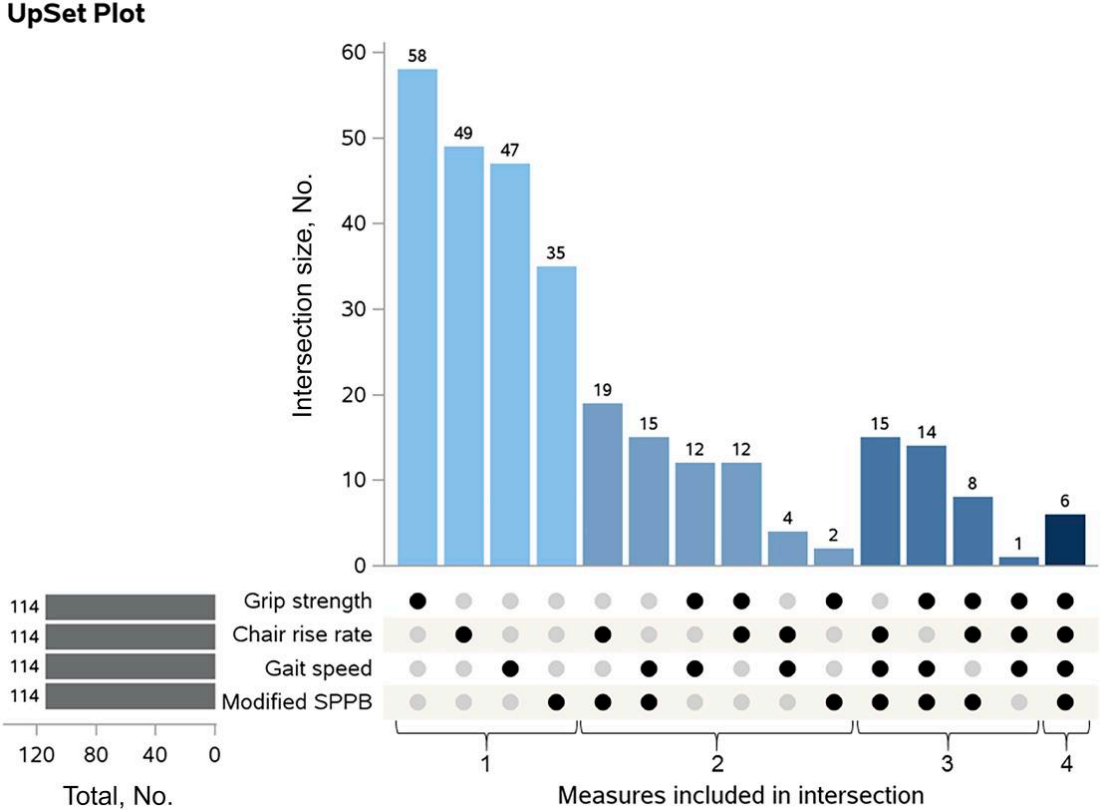
Overlap in measures of physical function decline among 669 participants with any decline. An UpSet plot visualizes overlap in physical function decline based on chair rise rate, gait speed, grip strength, and modified SPPB score. The bar chart on the left shows the total number of participants with decline according to each measure (n = 114 by definition). The bar chart on top shows the numbers of participants for each intersection of the 4 measures, shaded by the number of measures (from light for 1 measure to dark for all 4 measures); the measures represented in an intersection are shown with black circles in the legend below the chart; those not included are in gray. Abbreviation: SPPB, Short Physical Performance Battery.

The distributions of participants’ individual rates of change (per year) in each physical function measure are shown in Figure 1. Participants with an annualized rate of change indicating a decline of >0.35 rises/min/y in chair rise rate, >0.028 m/s/y in gait speed, >0.70 kg/y in grip strength or >0.032 on the mSPPB were classified to have physical function decline. With these criteria, 52% of participants had any decline in physical function; most participants had decline in 1 measure only (33%), 11.2% in 2 measures, 6.7% in 3, and only 1.1% (6 participants) had decline in all 4 measures (Figure 2).

### Factors Associated With Physical Function Decline

**Figure 3.**
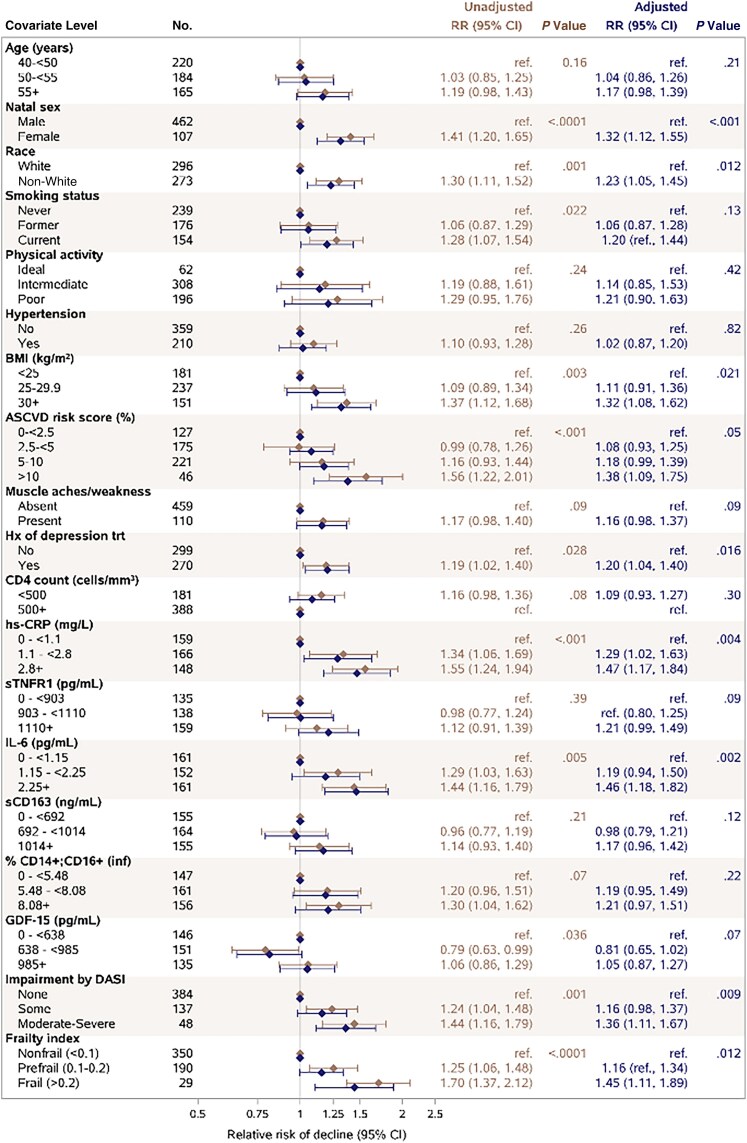
Baseline factors associated with physical function decline. Relative risk of physical function decline estimated from log binomial regression models with each factor evaluated individually (unadjusted models) and adjusted for age, race, and natal sex (adjusted models). Estimates are plotted in log scale for visual purposes. Abbreviations: ASCVD, Atherosclerotic Cardiac Risk Score; BMI, body mass index; DASI, Duke Activity Status Index; GDF-15, growth differentiation factor 15; hs-CRP, high-sensitivity C-reactive protein; IL-6, interleukin 6; RR, relative risk; sCD163, soluble CD163; sTNFR1, soluble tumor necrosis factor receptor–1.

In unadjusted models (Figure 3), we found a higher risk of physical function decline among females compared with males (RR, 1.41; 95% CI, 1.20–1.65; *P* < .0001) and non-White participants compared with White participants (RR, 1.30; 95% CI, 1.11–1.52, *P* = .001). Current smokers, those with higher BMI, history of depression treatment, higher ASCVD risk score, and levels of preexisting functional impairment or frailty also had a higher risk for physical function decline. Risk of decline increased with higher levels of hs-CRP (middle group: RR, 1.34; 95% CI, 1.06–1.69; high group: RR, 1.55; 95% CI, 1.24–1.94; *P* < .001), compared with the low hs-CRP group based on the tertiles, and IL-6 (RR, 1.29; 95% CI, 1.03–1.63; and RR, 1.44; 95% CI, 1.16–1.79, respectively; *P* = .005). GDF-15 also appeared associated with risk of decline but showed no consistent pattern across levels. While age effect did not reach statistical significance, the data suggest 19% higher risk of decline in the participants age 55 or older compared with those age 40–<50 years (95% CI, 0.98–1.43), with a similar estimated risk in those age 40–<50 and 50–<55 years. Other factors listed in the Methods showed no evidence of association with participant-specific changes in physical function measures in the screening based on Spearman correlation coefficients and were not included in the modeling analyses.

**Figure 4.**
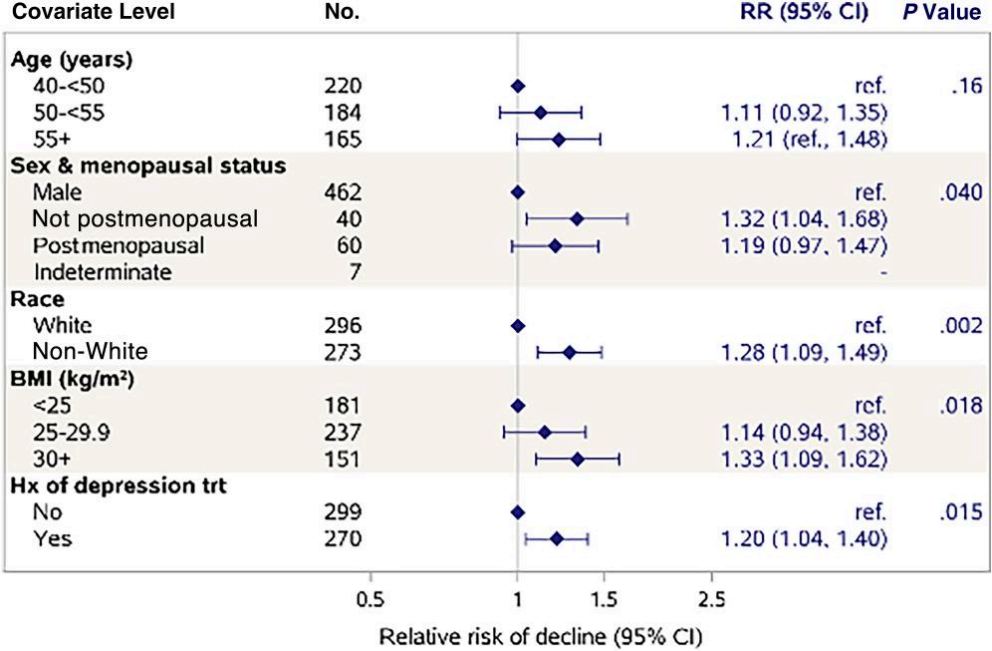
Risk of physical function decline by sex and menopausal status. Relative risk of physical function decline by sex and menopausal status estimated from a log binomial regression model adjusted for differences in characteristics between these groups (age, race, BMI, and history of depression treatment). Estimates are plotted in log scale for visual purposes. Abbreviations: BMI, body mass index; RR, relative risk.

In models adjusting for age, sex, and race (Figure 3), the effects of smoking status and ASCVD risk score were slightly attenuated, but the same trends remained apparent. The effects of other factors, except GDF-15, were robust to adjustment. To further explore whether the greater risk of decline among females (RR, 1.32 compared with males in the adjusted model) was driven by women who were postmenopause, we evaluated risk by menopausal status (Figure 4). The risk of decline was 19% higher among postmenopausal females (n = 60; RR, 1.19; 95% CI, 0.97–1.47) and 32% higher among pre- or perimenopausal females (n = 40; RR, 1.32; 95% CI, 1.04–1.68), both compared with males (*P* = .04 for any difference from males) (Figure 4). We further explored the sex difference in expanded models adjusting for BMI, history of depression treatment, and individual inflammatory markers (1-by-1), all of which were higher or more prevalent among female participants at baseline. The sex effect was attenuated in expanded models, particularly when adjusted for hs-CRP (RR, 1.16; 95% CI, 0.98–1.38; *P* = .09), but remained significant in models that included IL-6 (RR, 1.21; 95% CI, 1.02–1.44; *P* = .031), soluble CD-163 (RR, 1.20; 95% CI, 1.02–1.41; *P* = .029), and GDF-15 (RR, 1.18; 95% CI, 1.01–1.39; *P* = .041).

## DISCUSSION

In a large cohort of well-characterized PWH with low to moderate cardiovascular risk and objectively measured physical function over a median follow-up of nearly 5 years, we examined factors associated with physical function decline. Most participants had a decline in only 1 measure of physical function, with only 1% experiencing declines in all 4 assessed outcomes. We found higher risk of decline in physical function among those of non-White race and females; the data also suggested a higher risk in those 55 or older. The sex difference was observed in both pre- and perimenopausal women compared with men and was not fully explained by history of depression treatment, BMI, or level of inflammation, all found to be higher among females. After adjusting for sex, race, and age, we found that higher BMI, inflammation, levels of preexisting functional impairment and health deficits, and history of depression treatment were associated with greater risk of subsequent physical function decline. Identification of these factors, particularly modifiable factors such as BMI, can help clinicians provide early intervention strategies to prevent physical function decline.

Physical function decline was classified based on the observed 20th percentiles in the annualized rate of change, similar to how frailty is operationalized in large population studies [14]. Clinically meaningful differences are also commonly used to define physical function decline, indicating the change in patient perception of health status that corresponds with change in the performance measure [19]. The size of clinically meaningful differences varies according to the context, clinical outcome, and specific population of interest, and clinically meaningful differences are not available for all measures [19]. The data-driven approach we used facilitates identification of decreasing physical function in a relatively young and high-functioning population, where changes in physical function may be subtle and not immediately translate to changes in health status. Our observed thresholds for physical function decline are lower than clinically meaningful differences reported in the literature, which was expected given the different populations from which these differences were derived (ie, frail elderly, postsurgical) [20–22]. Among observational cohorts of people with HIV, longitudinal measurement of gait speed and grip strength have been previously published [6, 7]. In the Multicenter for AIDS Cohort Study (MACS), men with HIV had a similar decline in grip strength of 0.42 kg/y, compared with our reported threshold of grip strength decline of 0.41 kg/y. Greater gait speed decline in MACS participants (0.025 m/s/y compared with our threshold of 0.014 m/s/y) may be explained by greater comorbidity burden than participants in the present analysis. To our knowledge, this is the first analysis to quantify longitudinal change for chair rise and modified SPPB among those with HIV.

Our findings expand on prior work that has examined longitudinal physical function decline among PWH. Our findings that non-White participants, those with higher BMI, and those with a history of depression treatment have a greater risk of physical function decline are consistent with results from analyses in the MACS. Schrack et al. found that race and BMI were associated with declining gait speed and grip strength over 6 years [6, 7]. In a study examining the impact of diabetes on declining physical function among PWH, depression was found to be associated with worsening gait speed and grip strength over a mean follow-up time of 8.7 years [23]. Therefore, these subsets of individuals may particularly benefit from early screening and interventions to address the modifiable factors to prevent physical function decline.

The estimates of modifiable or preventable factors, such as BMI and depression treatment, were robust even when adjusted for age, race, and sex. Although the effects of ASCVD risk score and smoking status, other modifiable behaviors, were attenuated when adjusted for age, race, and sex, the effect estimates suggest increasing risk from 8% to 38% across ASCVD risk categories and a 20% higher risk among current smokers. Interventions to address these factors, particularly when implemented at this younger age (40–50 years old), may attenuate further functional decline and increase the likelihood of healthy aging later in life. Exercise and physical activity are an effective intervention that directly acts upon several of these factors (ie, decreases BMI and improves depressive symptoms) and improves physical function among PWH [24–26]. Pooled studies from the general adult population show that exercise is associated with a lower ASCVD risk factor score. Aerobic exercise bouts as short as 10 minutes can provide substantial benefit for lowering cardiovascular risk, provided that the total volume per week meets or exceeds 300 minutes [27, 28].

To our knowledge, ours is the first study to report sex differences in longitudinal physical function decline among PWH. Prior cross-sectional studies have shown poorer physical function among female compared with male PWH [29]. We similarly found that female participants had a greater risk of physical function decline compared with male participants. Baranoski et al. reported that 20% of female PWH had poor physical function as measured by the SPPB, and a low SPPB score was associated with a greater number of comorbidities [30]. Among older PWH in West Africa, females had a higher risk of low SPPB score compared with male participants [31]. An unexpected finding of our analysis was that the estimated sex difference was greater in the pre- and perimenopausal group than the postmenopausal group. In the Study of Women's Health Across the Nation (SWAN), postmenopausal women without HIV had increased odds of having a substantial limitation in self-reported physical function [32]; this association was attenuated but remained robust in models that included BMI and depressive symptoms [32]. One possible explanation for our finding that sex differences in physical function decline were most apparent in the comparison of women who had not yet undergone menopause vs men was that the premenopausal group may have been enriched with perimenopausal women.

Consistent with prior literature in people without HIV, we found that participants with higher levels of IL-6 and hs-CRP had greater risks of decline. Although other inflammatory markers did not reach statistical significance, the observed data were generally consistent with higher levels of inflammatory markers associated with higher risk for decline. This extends findings from cross-sectional studies of PWH that link higher inflammation with poor physical function. Erlandson et al. found that higher IL-6 levels were associated with increased odds for functional impairment measured by the SPPB and 400-meter walk [33]. Among women with HIV, Baranoski et al. reported that poor physical function on the SPPB was associated with higher IL-6 levels [30]. Finally, Derry et al. reported that inflammatory markers, including IL-6, were associated with poor self-reported physical function even after adjusting for age, sex, race, BMI, comorbidity burden, smoking status, and medication use among older adults with HIV [34]. Chronic inflammation is considered a hallmark of aging and of HIV pathogenesis and may be more prevalent among aging PWH due to side effects from ART, microbial translocation, and coinfections with other pathogens such as hepatitis C and cytomegalovirus [35]. Interventions to decrease inflammation may facilitate the preservation of physical function and healthy aging.

Several limitations of this analysis should be acknowledged. First, the population included in the analysis had limited female representation (19% female), there was no considerable representation of those identifying as a race other than White or Black, and all participants were enrolled in the United States, which limits the generalizability of the findings. This analysis included adults with HIV with low to moderate risk for cardiovascular disease by the ASCVD risk score per the REPRIEVE eligibility criteria, which may limit the generalizability of the findings to those with a higher comorbidity burden. Further, females and non-White participants with a worse clinical health status may have been eligible to enter, based on ASCVD risk score, which estimates that females and non-Whites have a lower risk of CVD. Finally, although some of the inflammatory markers were found to be predictive, overall baseline levels of inflammation were low. Future studies should examine whether early interventions can attenuate the physical function decline seen among PWH. This analysis also has several notable key strengths. The large representation of middle-aged adults with HIV provides information on factors associated with decline at an age where clinicians can effectively intervene. Interventions for modifiable factors, such as depression and high BMI, can be implemented at an earlier age. Midlife is a critical window to implement behaviors that will have long-lasting impacts on healthspan [36].

## CONCLUSIONS

We examined factors associated with physical function decline, defined as the annualized rate of change below the 20th percentile of the study population, in a large cohort (n = 569) of PWH in the United States. PWH who are female and non-White and have a higher BMI, preexisting functional impairment, or a history of depression treatment may be at greater risk for functional decline. Understanding factors associated with physical function decline can help identify those who may benefit from early intervention strategies to preserve function with aging.
